# Samuel H. Gray, M. D.

**Published:** 1885-10

**Authors:** 


### SAMUEL H. GRAY, M. D.

This good and true man has gone home. Another Christian martyr has sunk under the weight of arduous professional duties. The many friends of Dr. Gray will mourn his loss, not only as a useful member of society, but as an ornament of the medical profession. It may be truly said of him that he died at his post; exhausted with the paternal care of a beloved child, that could not be rescued by the devotion of his colleagues, he essayed to serve others with his remaining feeble powers, and became completely and irrevocably prostrated, falling in the harness August 31st, 1885.

The subject of this notice was born January 10th, 1846, near Bolingbroke, in Monroe county, Georgia, and joined the Methodist church at the early age of eleven years. His literary course was interrupted by the war, and he left Hilliard Institute, in Forsyth, Georgia, to join the army when only a youth, continuing in service until his command surrendered with Johnston's army. Subsequently, he was successfully employed in teaching, and entered upon the study of medicine, attending his first course in the Atlanta Medical College. He received his doctorate in 1867 from the Medical Department of the University of Georgia, at Augusta, and began practice in his native county in 1869. Dr. Gray proved himself to be a most acceptable physician amongst his old associates and friends, but a few years since changed his location to Barnesville, where his sphere of usefulness has been still more extended in his profession. His professional attainments and qualifications were of a high order; his genial temperament imparted a special fitness for the practice of medicine, so that he secured the confidence and affections of his patrons in his new home, as he had done among the friends of his youth in Monroe county. He was married in 1872 to Miss Lula Hollis, of Monroe county, who cheered his path in life, and by her gentle ministrations consoled him in his last illness, while she is left to mourn his loss. Being the eldest of five children, his parents, with two sisters and two brothers, remain crushed with the sad bereavement, but with the hope of a happy reunion in the world of spirits. His brothers, Dr. James A. Gray, the managing editor of this journal, and Rev. J. D. Gray, Presiding Elder in the North Georgia Conference, have the sympathy of a large circle of friends.

